# The impact of moderate and severe asthma exacerbations on quality of life: a post hoc analysis of randomised controlled trial data

**DOI:** 10.1186/s41687-020-00274-x

**Published:** 2021-01-12

**Authors:** Andrew Briggs, Shuaib Nasser, Eva Hammerby, Sarah Buchs, J. Christian Virchow

**Affiliations:** 1 Department of Health Services Research & Policy, London School of Hygiene & Tropical Medicine, London, England; 2 Department of Allergy, Cambridge University Hospitals NHS Foundation Trust, Cambridge, England; 3 ALK-Abello A/S, Bøge Allé 1, DK-2970 Hørsholm, Denmark; 4 Department of Pulmonology/Intensive Care Medicine, University of Rostock, Rostock, Germany

**Keywords:** Allergic asthma, Asthma, House dust mite allergic asthma, Asthma exacerbation, Exacerbation, Quality of life, Utility

## Abstract

**Background:**

This paper reports the duration of moderate and severe exacerbations in patients with house dust mite induced allergic asthma and the impact on patients’ quality of life.

**Methods:**

Post-hoc analyses were conducted using data collected during a phase III multi-national trial (MT-04) that investigated time to moderate or severe asthma exacerbation among 485 patients during withdrawal from inhaled corticosteroids. Patient diaries were analysed to ascertain duration of exacerbations. The impact on patients’ quality of life was measured by calculating utilities for five health states using the EuroQol-5 Dimension (EQ-5D-3L) and Asthma Quality of Life Questionnaire (AQL-5D). A regression analysis predicted the disutility of moving from ‘well controlled asthma’ to the other four health states: ‘partially controlled asthma’, ‘uncontrolled asthma’, ‘moderate exacerbation’ and ‘severe exacerbation’.

**Results:**

Two hundred four patients experienced exacerbations. Moderate and severe exacerbations involved statistically significant reductions in lung function compared to the constant peak expiratory flow observed for patients without exacerbations. Lung function decline occurred for 28 days, decreasing approximately 14 days before an exacerbation followed by a return to baseline over 14 days. Asthma symptoms, the use of short-acting β2-agonists, and frequency of nocturnal awakening all increased, starting 10–14 days before an exacerbation, and returned to baseline within 10–28 days following exacerbations. Compared to ‘well controlled asthma’, the disutility of having a ‘moderate exacerbation’ ranged from − 0.0834 to − 0.0921 (EQ-5D-3L) and from − 0.114 to − 0.121 (AQL-5D); and of having a ‘severe exacerbation’ from − 0.115 to − 0.163 (EQ-5D-3L) and from − 0.153 to − 0.217 (AQL-5D), depending on the length of the observation period.

**Conclusions:**

The impact of moderate and severe exacerbations in house dust mite induced allergic asthma extends 14 days before and 28 days after the peak exacerbation event. The impact of exacerbations on patients’ health-related quality of life (HRQoL) continues long after their occurrence.

## Background section

Allergic asthma (AA) is the most common type of asthma [1]. It is a chronic global health problem accounting for substantial morbidity and health-care expenditures [2, 3]. The total annual cost of asthma in Europe is estimated to be €19.3 billion [4]. Moderate and severe exacerbations significantly increase asthma-related and total health-care costs [5].

House dust mite (HDM) induced AA (HDM AA) is a hypersensitivity reaction to inhaled airborne HDM allergens. HDM is the most prevalent indoor allergen associated with asthma [6, 7], with 47.7% of European adults with asthma sensitised to HDM allergens [8]. HDM AA adversely impact patients’ health-related quality of life (HRQoL), both through asthma symptoms and exacerbations [9–12].

Exacerbations are serious complications of asthma, characterised by episodes of acute deterioration of progressively worsening bronchial obstruction, which can lead to shortness of breath, coughing, wheezing and/or chest tightness [13]. Exacerbations may subside spontaneously or in response to treatment, but often require systemic corticosteroids for 5 to 7 days [11]. Severe exacerbations may require hospitalisation and can be life-threatening [11, 14], and while they are more common with poorly controlled asthma [15], they can also occur in mild [16, 17] or well-controlled asthma [18].

The Global Initiative for Asthma (GINA) outlines the characteristics that determine whether patients are classified as having ‘well controlled’, ‘partly controlled’ or ‘uncontrolled’ asthma: daytime symptoms, limitations of activities, nocturnal symptoms/awakening, need for reliever/rescue treatment, lung function and exacerbations [19]. GINA recommends [19] that a patient’s level of asthma control (their GINA “control status”) and current treatment should determine what pharmacologic treatment should be selected.

The only disease-modifying treatment for allergic disease including AA is allergy immunotherapy (AIT). AIT has been shown to limit disease progression in children with HDM allergy by preventing the development of new allergic sensitisations [20]. HDM sublingual immunotherapy (SLIT) is recommended in the GINA guidelines as a treatment for uncontrolled asthma with exacerbations despite inhaled corticosteroids (ICS) provided the FEV_1_ is > 70% predicted [21].

The multinational European MT-04 trial was the first trial to assess asthma exacerbations using AIT [22]. Although exacerbations are a key outcome in asthma research, trials often do not distinguish between moderate and severe exacerbations [23]. Lloyd [12] investigated the impact of exacerbations on HRQoL by different severity grades of exacerbations, defined by what treatment was required to treat the exacerbation. MT-04 was the first study to include an assessment of both moderate and severe exacerbations using the following definitions: the ATS/ERS definition of a moderate exacerbation translated into a more measurable format suitable for a clinical trial [24]; and the ATS/ERS description for severe exacerbations [13] (Table 1).

**Table 1:** Definitions of moderate and severe exacerbations used in the MT-04 trial

Criteria defining moderate exacerbations [24]	Criteria defining severe exacerbations [13]
1 or more of the following criteria and must also result in a temporary change in treatment: ● Nocturnal awakening(s) due to asthma requiring SABA use for at least 2 consecutive nights or an increase of at least 0.75 points in daily symptom score from baseline value on at least 2 consecutive days; ● An increase from baseline in SABA use on at least 2 consecutive days (a minimum increase of 4 puffs per day); ● A 20% or more decrease in peak expiratory flow from baseline on at least 2 consecutive mornings or evenings or a 20% or more decrease in FEV_1_ from baseline; ● A visit to the emergency department or an unscheduled visit to the trial site for asthma treatment not requiring systemic corticosteroids.	1 or more of the following criteria: ● A requirement for systemic corticosteroids for the treatment of asthma symptoms for at least 3 days; ● An emergency department visit due to asthma requiring systemic corticosteroids, or a hospitalization for more than 12 h due to asthma.

*FEV*_*1*_ Forced Expiratory Volume in 1 s, *SABA* Short-acting β_2_-agonists

The current paper reports post-hoc analyses of the MT-04 trial data that investigated the duration of both moderate and severe exacerbations and their impact on patients’ HRQoL, measured through utility. This is of relevance as only limited data are available about exacerbation duration, with one patient survey suggesting that this could be more than 21 days [23].

## Methods

### Study subjects

The MT-04 trial methods have been described in detail elsewhere [22]. The relevant independent ethics committees and institutional review boards approved the trial protocol and amendments in each of the 13 countries, and the trial followed the principles of the Helsinki Declaration.

### Study design

This paper reports two post-hoc analyses of the European MT-04 phase III multi-national, randomised, double-blind, placebo-controlled trial. The primary endpoint of MT-04 was time to first moderate or severe asthma exacerbation during the period of ICS reduction and withdrawal. The first post-hoc analysis investigated the duration of exacerbations through an analysis of patients’ electronic diaries (e-diaries). The second analysis derived utilities (patients’ preferences) for five mutually exclusive asthma health states (well controlled, partially controlled, uncontrolled, moderate and severe exacerbation) derived from the trial data through a stepwise approach in order to measure the impact of asthma control and exacerbations on patients’ HRQoL.

### Methods

**Fig. 1:**
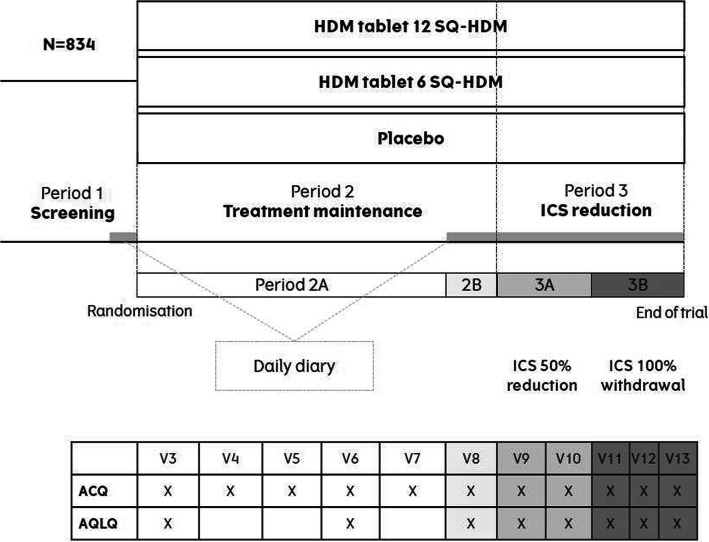
MT-04 trial design

The MT-04 trial was divided into three periods (see Fig. 1). In period 1 (5 to 7 weeks), patients were screened and switched from their regular ICS asthma controller to budesonide and SABA. Following randomisation to either SQ® HDM SLIT-tablet (6 SQ-HDM or 12 SQ-HDM) or placebo, patients continued with treatment during period 2 (7–12 months). In period 3, treatment with SQ® HDM SLIT-tablet and placebo continued but patients’ daily ICS was reduced by 50% for 3 months (period 3A) and was then withdrawn for the next 3 months (period 3B).

The following data, relevant for the present post-hoc analysis, were collected:

- E-diaries, capturing asthma symptoms (measured using the Daily Symptom Score), SABA use (puffs per day), nocturnal awakenings (times per night) and lung function (PEF), were completed twice daily, starting during the final 4 weeks of period 2 (defined as period 2B) and continuing throughout period 3 (3A and 3B).
- Asthma Control Questionnaire (ACQ-7) data, assessing patients’ asthma control on a 7-point scale (0 = no impairment, 6 = maximum impairment) across 7 domains (the top scoring 5 symptoms, FEV_1_% predicted value and daily SABA use), were collected at 11 scheduled visits during periods 2 and 3.
- The Asthma Quality of Life Questionnaire (AQLQ) measuring the functional problems (in physical, emotional, social and occupational domains) that are most troublesome to adults with AA was completed by patients once during the middle of period 2 and five times throughout period 3 (including at unscheduled visits due to exacerbations) (Fig. 1). The AQLQ uses a 7-point polytomous response scale ranging from 1 (severely impaired) to 7 (not impaired at all) across the four domains.

### Analysis

The post-hoc analyses investigated the duration of moderate and severe exacerbations through patient diary data, and the impact of exacerbations on patients’ HRQoL by using AQLQ data to derive utilities.

### Duration of exacerbations

E-diary data was used to visualise the duration of exacerbations. Baseline-adjusted mean scores for e-diary data for the 4 weeks before and after the patients’ first exacerbation were compared to the final 8 weeks of data for patients who did not experience exacerbations. For subjects who did not exacerbate, the last 8 weeks of e-diary data were used as this period (3B, ICS withdrawal) is when many exacerbations occur and was expected to provide the most appropriate control group.

### Deriving utilities

**Fig. 2:**
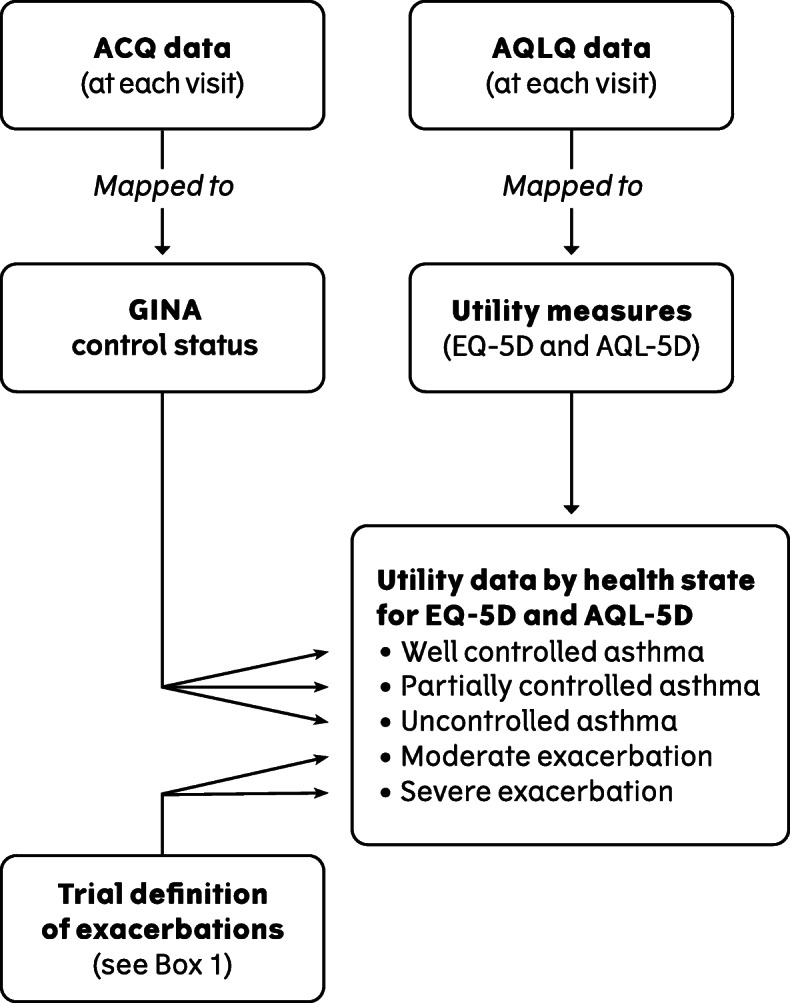
Overview of analysis conducted on the MT-04 trial data

Utilities at each visit for 5 mutually exclusive asthma health states were derived by mapping AQLQ data (Fig. 2) using the definition of asthma exacerbations used in the trial (Table 1) and GINA asthma control status. AQLQ data for each patient was categorized into the health states in a hierarchical approach at each scheduled or unscheduled visit. If patients had an exacerbation within a given number of days after a visit, the AQLQ data was categorized as a moderate or severe exacerbation at that visit. All remaining AQLQ data points were grouped according to the GINA asthma control status of the patient. Categorisation of control status was done by mapping ACQ data to the GINA asthma control status categories (Fig. 2) (well controlled, partially controlled and uncontrolled) using a published algorithm [25]. Observation periods of 7, 14, 21 and 28 days from an asthma exacerbation were used to include AQLQ data, in order to explore how long the impact of an asthma exacerbation on patients’ utility lasts.

Utility values were obtained in two ways: by mapping to the Asthma Quality of Life Questionnaire (AQL-5D) from AQLQ; and by mapping to the 3-level version of the EuroQol-5 Dimension (EQ-5D-3L) from AQLQ. The AQL-5D is derived from the AQLQ, a disease-specific questionnaire, and can be used in economic evaluation. It was decided to also map to EQ-5D-3L since data derived using the EQ-5D-3L is generally preferred by health technology assessment agencies when undertaking economic evaluation. The two questionnaires use different value sets to calculate the utility data, which results in similar, but not identical values.

The mapping from AQLQ to EQ-5D-3L and AQL-5D was done using previously developed algorithms [26, 27]. The mapping produced utility values for each patient at each scheduled and unscheduled visit in the trial. These utilities were entered into a mixed effects ML regression analysis, which estimated the predicted utility for well controlled asthma, and the predicted disutility of moving from well controlled asthma to the four health states of partially controlled asthma, uncontrolled asthma, moderate exacerbation and severe exacerbation’ (Fig. 2). The model of estimated EQ-5D-3L and AQL-5D can be seen in Supplement 1.

## Results

### MT-04 patient details and asthma control

Seven hundred forty-two patients completed the trial and were included in the full analysis set [22]. Four hundred eighty-five patients received the active treatment (237 6 SQ-HDM SLIT, 248 12 SQ-HDM SLIT) and 257 received a placebo. The patient baseline characteristics and patient discontinuations are reported elsewhere [22]. At randomisation the percentage of patients reported to have partly controlled asthma was similar in the two groups (active treatment: 71%; placebo: 72%).

### Duration of exacerbations

**Fig. 3:**
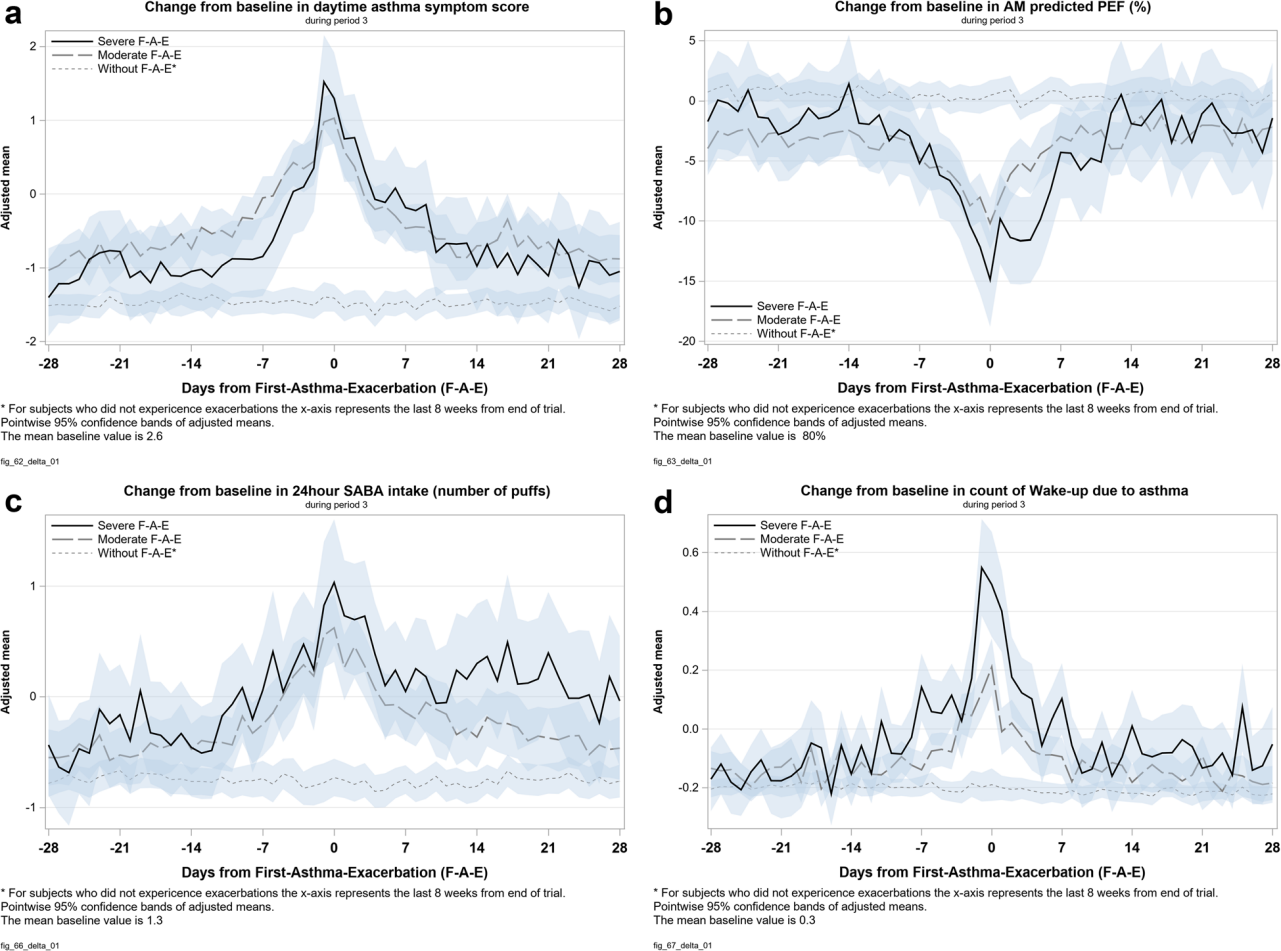
Duration of exacerbations

Two hundred four patients experienced a moderate or a severe exacerbation during period 3 of the trial. E-diary data demonstrated that moderate and severe exacerbations involved statistically significant reductions in lung function (morning and evening) compared to the average constant peak expiratory flow (PEF) observed for patients who did not experience exacerbations (Fig. 3). For both moderate and severe exacerbations lung function reduction occurred for about 28 days and started to decrease approximately 14 days before an exacerbation followed by a gradual return to baseline over 14 days.

During both moderate and severe exacerbations, asthma symptoms, SABA use, and the frequency of nocturnal awakening all increased, starting 10–14 days before an exacerbation and returned to baseline within 10–28 days following the exacerbation (Fig. 3).

There was a statistically significant difference in symptoms between those with moderate or severe exacerbations compared to patients who did not experience exacerbations (*p* < 0.05).

### Utilities

Utility values were derived for five asthma health states: well controlled, partially controlled, uncontrolled, moderate and severe exacerbation (Table 2).

**Table 2:**
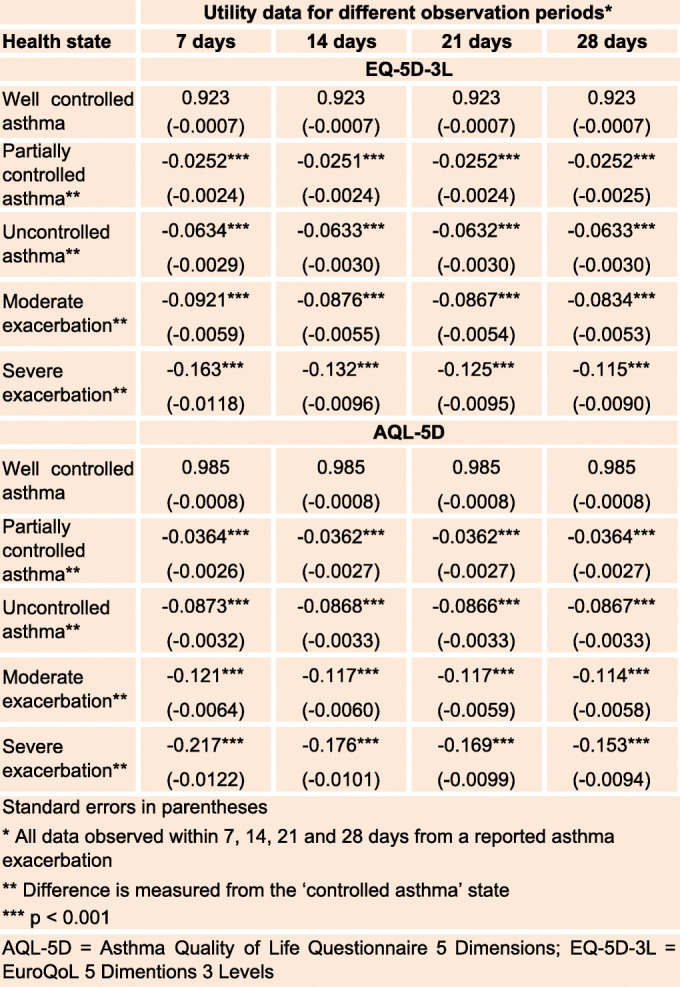
Utilities by asthma health state

The utility values for the ‘well controlled asthma’ health state were 0.923 (standard error (SE): 0.000716) for EQ-5D-3L and 0.985 (SE: 0.000844) for AQL-5D. The disutility values of having ‘partially controlled asthma’ compared to ‘well controlled asthma’, for each of the four cut-offs for the number of days, ranged from − 0.0251 to − 0.0252 for EQ-5D-3L and − 0.0362 to − 0.0364 for AQL-5D; and of having ‘uncontrolled asthma’ compared to ‘well controlled asthma’, ranged from − 0.0632 to − 0.0634 for EQ-5D-3L and − 0.0866 to − 0.0873 for AQL-5D.

Compared to ‘well controlled asthma’, the data for disutility of having a ‘moderate exacerbation’ ranged from − 0.0834 to − 0.0876 for EQ-5D-3L and − 0.114 to − 0.121 for AQL-5D; and of having a ‘severe exacerbation’ ranged from − 0.115 to − 0.163 for EQ-5D-3L and − 0.153 to − 0.217 for AQL-5D. Compared to ‘well controlled asthma’, the disutility values of having exacerbations were more than having partially controlled or uncontrolled asthma.

## Discussion

This post-hoc analysis demonstrates that the impact of moderate and severe exacerbations in HDM AA extends long before and after the peak exacerbation event. Symptoms and medication use increase up to 14 days prior to the onset of an exacerbation. After the exacerbation, patients’ symptoms and medication use returns to baseline more slowly than reported in the literature [28]. For example, lung function took 14 days on average to return to baseline and patients’ asthma symptoms, SABA use and frequency of nocturnal awakening took between 10 and 28 days to return to baseline. This demonstrates that PEF and symptomatic measures describe different aspects of asthma control and might not be useful as proxies. Patients’ symptoms and medication use are also affected for 14 days after an exacerbation.

Similarly, the impact of exacerbations on HRQoL (utility) continues long after the occurrence of the exacerbations. Not only do utility scores exhibit change over several weeks around an exacerbation, but the size of the change is also important. These analyses showed that AA patients experience a reduction in HRQoL (disutility) that is greater than 0.05 during moderate (range − 0.0834 to − 0.121) and severe (range − 0.115 to − 0.217) exacerbations, as well as during times of uncontrolled asthma (range − 0.0632 to − 0.0873). The disutilities obtained by mapping to AQL-5D were larger than those obtained by mapping to EQ-5D. This may be due to the AQL-5D being a disease-specific questionnaire and is therefore more sensitive to the impact on subjects’ HRQoL.

MT-04 demonstrated that time to a first moderate or severe exacerbation increases in patients who receive active treatment [22]. Prolonging the time to exacerbation means that patients experience a higher HRQoL for a longer period of time and experience fewer symptoms and require less reliever medication. The data for MT-04 are consistent with data reported by Briggs [29] who incorporated AQLQ data (from the Gaining Optimal Asthma control (GOAL) study mapped to utility) to analyse the cost-effectiveness of asthma control treatment. Briggs calculated a disutility of − 0.216 for exacerbations, but did not distinguish between moderate and severe. The definition of an exacerbation used in Briggs was similar to that for ‘severe’ exacerbation used in MT-04. The largest disutility value calculated for MT-04 was − 0.217 for a severe exacerbation. Lloyd [12] reported that EQ-5D was significantly worse for asthma patients experiencing exacerbations. They reported the utility of patients not in exacerbation was 0.89, the utility of patients in exacerbation without steroids was 0.57 and the utility of patients in exacerbation and hospitalised was 0.33. Although the patients in this trial had more severe asthma than those in MT-04, the impact of exacerbations on HRQoL in Lloyd et al. is similarly apparent.

The effect of asthma exacerbations on utilities is greater in MT-04 than the disutilities for acute events in other diseases. Harris [30] reported that non-severe hypoglycaemia resulted in HRQoL decreases of between − 0.0056 and − 0.0076 and decreases for severe hypoglycaemia between − 0.0592 and − 0.0616. Davies [31] reported disutilities for two acute events of − 0.06 for myocardial infarction and − 0.05 for unstable angina. This analysis of MT-04 has shown that the effects of asthma exacerbations can be experienced for weeks before and after the event, whereas events in other disease areas can be much shorter. For example, prolonged angina lasts more than 20 min [32], typical chest pain in acute myocardial infarction may last between 30 and 60 min [33] and the duration of a hypoglycaemic episode may be minutes or hours [34].

A limitation of this study is that the definition of a moderate asthma exacerbation was used for the first time in the MT-04 trial and might have limited usability in clinical practice. The number of exacerbations may be considered small. This is a result of the trial design, where patients were able to discontinue after the experience of the first asthma exacerbation or continue on an increased ICS dose. This approach may be the reason that few patients experienced more than one exacerbation. For the current analysis, this means that the impact of repeated exacerbations on HRQoL could not be measured. A further limitation was that the analyses did not control for the timing between exacerbation occurrence and AQLQ assessment. Finally, most of the trial analyses described in this paper were post-hoc.

Future research in AIT should carefully consider the method by which HRQoL is measured during exacerbations so that the impact of exacerbations can be captured accurately in terms of intensity as well as duration. Future modelling could analyse the relationship between utility and duration of exacerbation. HRQoL should also be measured in an AIT trial without ICS reduction and a primary endpoint which assesses the frequency of exacerbations rather than time to exacerbation. This would enable assessment of the impact on HRQoL and the health economic impact of repeated exacerbations. Future asthma research should seek to capture data on moderate exacerbations, to increase our understanding of their effects compared to severe exacerbations.

## Conclusions

In conclusion, moderate and severe HDM AA exacerbations lead to increases in symptoms and medication use as well as reduction in lung function over a longer time period than previously appreciated: up to 14 days prior to an exacerbation and up to 28 days after [28]. Exacerbations, both moderate and severe, lead to a decrease in HRQoL. Severe exacerbations lead to significantly more variation in lung function, asthma symptoms, SABA use and frequency of nocturnal awakening than moderate exacerbations.

## Supplementary Information

**Additional file 1: Table S1.** Models of estimated EQ-5D-3L and AQL-5D (All data observed within 7, 14, 21 and 28 days from a reported asthma exacerbation).

## Data Availability

The datasets used and analysed during the current study are available from the corresponding author on reasonable request.

## Abbreviations

AA: Allergic Asthma
ACQ: Asthma Control Questionnaire
AIT: Allergy Immunotherapy
AQL-5D: Asthma Quality of Life Questionnaire – 5 Dimensions
AQLQ: Asthma Quality of Life Questionnaire
ATS: American Thoracic Society
EQ-5D: Euroqol 5 Dimensions
ERS: European Respiratory Society
FEV1: Forced Expiratory Volume in 1 Second
GINA: Global Initiative for Asthma
GOAL: Gaining Optimal Asthma Control
HDM: House Dust Mite
HDM AA: House Dust Mite induced Allergic Asthma
HRQoL: Health-relatedQuality of Life
ICS: Inhaled Corticosteroid
PEF: Peak Expiratory Flow
SABA: Short-Acting Beta Agonists
SLIT: Sublingual Immunotherapy
TTO: Time Trade-Off
