# Supplementary material for: The impact of moderate and severe asthma exacerbations on quality of life: a post hoc analysis of randomised controlled trial data
Source: J Patient Rep Outcomes. 2021 Jan 12;5:6. doi: 10.1186/s41687-020-00274-x (PMC7803866; doi:10.1186/s41687-020-00274-x)
Supplement: Supplementary file 1 — Additional file 1: Table S1. Models of estimated EQ-5D-3L and AQL-5D (All data observed within 7, 14, 21 and 28 days from a reported asthma exacerbation). [file 41687_2020_274_MOESM1_ESM.docx]

Supplement 1

**Supplementary Table 1: Models of estimated EQ-5D-3L and AQL-5D (All data observed within 7, 14, 21 and 28 days from a reported asthma exacerbation)**

|  | AQL-5D | | | | Estimated EQ-5D-3L | | | |
| --- | --- | --- | --- | --- | --- | --- | --- | --- |
|  | ≤ 7 | ≤ 14 | ≤ 21 | ≤ 28 | ≤ 7 | ≤ 14 | ≤ 21 | ≤ 28 |
| main |  |  |  |  |  |  |  |  |
| Partial control | -0.0364^***^ (0.00263) | -0.0362^***^ (0.00267) | -0.0362^***^ (0.00268) | -0.0364^***^ (0.00269) | -0.0252^***^ (0.00241) | -0.0251^***^ (0.00244) | -0.0252^***^ (0.00244) | -0.0252^***^ (0.00245) |
|  |  |  |  |  |  |  |  |  |
| Uncontrolled | -0.0873^***^ (0.00321) | -0.0868^***^ (0.00326) | -0.0866^***^ (0.00326) | -0.0867^***^ (0.00328) | -0.0634^***^ (0.00293) | -0.0633^***^ (0.00297) | -0.0632^***^ (0.00297) | -0.0633^***^ (0.00298) |
|  |  |  |  |  |  |  |  |  |
| Moderate exacerbation | -0.121^***^ (0.00636) | -0.117^***^ (0.00597) | -0.117^***^ (0.00585) | -0.114^***^ (0.00575) | -0.0921^***^ (0.00594) | -0.0876^***^ (0.00550) | -0.0867^***^ (0.00538) | -0.0834^***^ (0.00529) |
|  |  |  |  |  |  |  |  |  |
| Severe exacerbation | -0.217^***^ (0.0122) | -0.176^***^ (0.0101) | -0.169^***^ (0.00992) | -0.153^***^ (0.00937) | -0.163^***^ (0.0118) | -0.132^***^ (0.00964) | -0.125^***^ (0.00945) | -0.115^***^ (0.00899) |
|  |  |  |  |  |  |  |  |  |
| Baseline AQL-5D | 0.419^***^ (0.0159) | 0.419^***^ (0.0161) | 0.419^***^ (0.0161) | 0.418^***^ (0.0161) |  |  |  |  |
|  |  |  |  |  |  |  |  |  |
| Baseline estimated EQ-5D-3L |  |  |  |  | 0.449^***^ (0.0151) | 0.450^***^ (0.0152) | 0.450^***^ (0.0152) | 0.449^***^ (0.0153) |
|  |  |  |  |  |  |  |  |  |
| Constant | 0.593^***^ (0.0146) | 0.593^***^ (0.0147) | 0.592^***^ (0.0147) | 0.594^***^ (0.0147) | 0.524^***^ (0.0132) | 0.523^***^ (0.0133) | 0.523^***^ (0.0133) | 0.524^***^ (0.0134) |
| lns1_1_1 |  |  |  |  |  |  |  |  |
| Constant | -3.341^***^ (0.0366) | -3.334^***^ (0.0367) | -3.333^***^ (0.0367) | -3.335^***^ (0.0368) | -3.475^***^ (0.0375) | -3.467^***^ (0.0375) | -3.467^***^ (0.0375) | -3.466^***^ (0.0376) |
| lnsig_e |  |  |  |  |  |  |  |  |
| Constant | -2.813^***^ (0.0102) | -2.798^***^ (0.0102) | -2.798^***^ (0.0102) | -2.794^***^ (0.0102) | -2.921^***^ (0.0104) | -2.912^***^ (0.0104) | -2.911^***^ (0.0104) | -2.908^***^ (0.0104) |
| Observations | 5615 | 5633 | 5633 | 5633 | 5364 | 5379 | 5379 | 5379 |
| *AIC* | -14674.7 | -14565.0 | -14557.0 | -14521.2 | -15191.3 | -15134.9 | -15125.8 | -15102.7 |

Standard errors in parentheses

^*^ *p* < 0.05, ^**^ *p* < 0.01, ^***^ *p* < 0.001

AIC = Akaike Information Criterion; AQL-5D = Asthma Quality of Life Questionnaire – 5 Dimensions; EQ-5D-3L = EuroQoL Five-Dimensional Questionnaire 3 Levels
